# Intra-Operative Electron Radiation Therapy (IOERT) Anticipated Boost in Breast Cancer Treatment: An Italian Multicenter Experience

**DOI:** 10.3390/cancers14020292

**Published:** 2022-01-07

**Authors:** Antonella Ciabattoni, Fabiana Gregucci, Karen Llange, Marina Alessandro, Francesca Corazzi, Giovanni B. Ivaldi, Paola Zuccoli, Antonio Stefanelli, Agostino Cristaudo, Vincenzo Fusco, Loredana Lapadula, Alba Fiorentino, Daniela Di Cristino, Francesca Salerno, Marco Lioce, Marco Krengli, Cristiana Vidali

**Affiliations:** 1 Department of Radiation Oncology, Ospedale San Filippo Neri, ASL Roma 1, 00135 Rome, Italy; antonella.ciabattoni@aslroma1.it (A.C.); daniela.dicristino@aslroma1.it (D.D.C.); francesca.salerno@aslroma1.it (F.S.); 2 Department of Radiation Oncology, Ospedale Regionale Generale “F. Miulli”, 70021 Bari, Italy; a.fiorentino@miulli.it; 3 Department of Radiation Oncology, Hospice Carlo Chenis, ASL Roma 4, 00053 Roma, Italy; karen.llange@aslroma4.it; 4 Department of Radiation Oncology, Ospedale di Cittá di Castello, 06012 Perugia, Italy; marina.alessandro@uslumbria1.it (M.A.); francesca.corazzi@uslumbria1.it (F.C.); 5 Department of Radiation Oncology, ICS Maugeri SpA SB-IRCCS, 27100 Pavia, Italy; giovannibattista.ivaldi@fsm.it (G.B.I.); paola.zuccoli@fsm.it (P.Z.); 6 Department of Radiation Oncology, Arcispedale Sant’Anna, Azienda Ospedaliera Universitaria di Ferrara, 44121 Ferrara, Italy; a.stefanelli@ospfe.it (A.S.); agostino.cristaudo@ospfe.it (A.C.); 7 Department of Radiation Oncology, IRCCS CROB Rionero in Vulture, 85028 Potenza, Italy; vincenzo.fusco@crob.it (V.F.); lapa.dula@alice.it (L.L.); 8 Department of Radiation Oncology, Istituto Tumori “Giovanni Paolo II”, 70124 Bari, Italy; m.lioce@oncologico.bari.it; 9 Department of Radiation Oncology, Azienda Ospedaliera Universitaria Maggiore della Carità, 28100 Novara, Italy; marco.krengli@med.unipmn.it; 10 Department of Radiation Oncology, Azienda Sanitaria Universitaria Integrata di Trieste, 34142 Trieste, Italy; cristiana.vidali@libero.it

**Keywords:** early-stage breast cancer, whole breast irradiation (WBI), Intra-Operative Electron Radiation Therapy (IOERT), anticipated IOERT-boost, local recurrence, cosmetic result

## Abstract

**Simple Summary:**

In breast cancer, IOERT represents an attractive therapeutic option to deliver a boost directly on the tumor bed during surgery with all the relative radiobiological advantages. According to the literature, the present multicenter research demonstrates the safety and efficacy of this treatment using a large group of patients treated in daily clinical practice. Between January 2011 and March 2018, 797 patients were evaluated. Median follow-up was 57 months (range 12–109 months). Acute toxicity ≥ G2 occurred in 179 patients (22.46%). No patients reported late toxicity > G2. Obj-cosmetic result was excellent in 45%, good in 35%, fair in 20% and poor in 0% of cases. Subj-cosmetic result was excellent in 10%, good in 20%, fair in 69% and poor in 0.3% of cases. At 5 years, in-field LC was 99.2% (95% CI: 98–99.7); out-field LC 98.9% (95% CI: 97.4–99.6); DFS 96.2% (95% CI: 94.2–97.6); OS 98.6% (95% CI: 97.2–99.3).

**Abstract:**

In breast cancer, the use of a boost to the tumor bed can improve local control. The aim of this research is to evaluate the safety and efficacy of the boost with intra-operative electron radiotherapy (IOERT) in patients with early-stage breast cancer undergoing conservative surgery and postoperative whole breast irradiation (WBI). The present retrospective multicenter large data were collected between January 2011 and March 2018 in 8 Italian Radiation Oncology Departments. Acute and late toxicity, objective (obj) and subjective (subj) cosmetic outcomes, in-field local control (LC), out-field LC, disease-free survival (DFS) and overall survival (OS) were evaluated. Overall, 797 patients were enrolled. IOERT-boost was performed in all patients during surgery, followed by WBI. Acute toxicity (≥G2) occurred in 179 patients (22.46%); one patient developed surgical wound infection (G3). No patients reported late toxicity ≥ G2. Obj-cosmetic result was excellent in 45%, good in 35%, fair in 20% and poor in 0% of cases. Subj-cosmetic result was excellent in 10%, good in 20%, fair in 69% and poor in 0.3% of cases. Median follow-up was 57 months (range 12–109 months). At 5 years, in-field LC was 99.2% (95% CI: 98–99.7); out-field LC 98.9% (95% CI: 97.4–99.6); DFS 96.2% (95% CI: 94.2–97.6); OS 98.6% (95% CI: 97.2–99.3). In conclusion, IOERT-boost appears to be safe, providing excellent local control for early-stage breast cancer. The safety and long-term efficacy should encourage use of this treatment, with the potential to reduce local recurrence.

## 1. Introduction

Breast cancer is the most common malignant disease among women, and it still represents the leading cause of death from cancer among them (28% in patients under 50 years, 21% in those between 50 and 69 years, and 14% in those older than 70), even though there has been a downward trend in the mortality (0.8%/year) in recent years, due to the spread of screening programs, early diagnosis and therapeutic progress [1,2]. The standard treatment for early breast cancer includes breast conserving surgery, such as quadrantectomy, wide excision or lumpectomy, plus sentinel lymph node biopsy or, if necessary, axillary lymph node dissection, followed by postoperative whole breast irradiation (WBI) ± adjuvant systemic treatment.

Adjuvant WBI is currently considered the standard treatment after breast conservative surgery and plays an important role to reduce local recurrences (LR) and to improve disease-free survival (DFS) and overall survival (OS) [3].

Over the years, the most widely used dose was 50–50.4 Gy in daily fractions of 2–1.8 Gy in 5–5.5 weeks, generally using two fields tangent to the chest wall with or without a boost with either electrons or photons or brachytherapy to the tumor bed, which is regarded as the site with the highest probability of local recurrence.

In recent years, moderate hypofractionated WBI has become the preferred dose-fractionation scheme, generally using a total dose of 40 Gy in 15 fractions or 42.50 Gy in 16 fractions. The LR rate turned out to be even superior to that of the conventional schedule, while maintaining equal or even better long-term cosmetic results and toxicity [4,5].

The use of an additional external boost of 10–16 Gy to the tumor bed can reduce the local failure rate; this effect could be observed in all age classes, whereas the absolute gain is greatest in the group below 40 years. Adequate local control has been shown to confer a survival benefit at long-term follow-up [6].

Intra-operative radiation therapy (IORT) is a high-dose, single-session partial breast irradiation technique performed during breast-conserving surgery, and it is indicated either as an exclusive treatment in a subgroup of patients considered at low risk for LR [7] or as an anticipated boost to the tumor bed followed by WBI [8,9]_._

Direct irradiation during surgery has some evident advantages: the visualization of the tumor bed reduces the risk of a geographic miss while completely sparing the skin, which is an organ at risk for cosmetic late effects; moreover, reconstructive oncoplastic surgery, which has a potential to displace the true position of the tumor bed, hence bearing an additional risk of misalignment, does not exclude intra-operative irradiation, as it is performed prior to oncoplastic intervention [10]. In addition, it was hypothesized that it has implications on the tumor microenvironment, inhibiting the proliferative cascade induced by surgical wound healing. Another potentially positive aspect of intra-operative radiotherapy is the prevention of possible residual tumor cell repopulation between surgery and adjuvant radiotherapy. Furthermore, a good oxygenation status of the tumor bed during surgery could be a factor for enhanced biological effectiveness, which has not yet been investigated [11,12,13].

Two different IORT techniques are currently used: the low-kV photon system (e.g., Intrabeam) and the electron system produced by standard or “dedicated” mobile LINACs. Three different dedicated mobile electrons LINACs machines are commercially available: Liac HWL and Novac (S.I.T. Sordina IORT Technologies S.p.A., Vicenza, Italy) and Mobetron (IntraOp Medical Corporation, Sunnyvale, CA, USA) [14]. At the moment, the largest evidence for boost IORT originates from electrons (IOERT) [9,15].

The anticipated IOERT-boost is delivered at doses of 9–12 Gy, prescribed at a 90–100% isodose followed by WBI to a total dose of 50 Gy in 25 fractions (fr) or radio-biologically equivalent doses (40.5 Gy/15 fr or 42.56 Gy/16 fr) [8,16].

A minimum interval of approximately 4 weeks is recommended between IOERT and external beam radiotherapy (EBRT), provided the surgical wound has healed well [9].

Based on this background, the aim of this multicenter Italian retrospective analysis was to confirm, in daily clinical practice, the safety and effectiveness of the IOERT-boost treatment in a large study population of patients with early-stage breast cancer in terms of toxicity, cosmetic outcome, local/distant control and survival.

## 2. Materials and Methods

Retrospective data were collected from a total of 797 patients affected by early-stage breast cancer treated with conservative surgery, IOERT-boost and WBI from January 2011 to March 2018 in 8 Italian Radiotherapy Centers (Table 1). Two centers (Trieste and Novara) used the Mobetron, whereas the others used the Novac and Liac dedicated LINACs.

Regarding conservative surgery, all patients underwent quadrantectomy with a free margin of at least 10 mm. Patients with positive margins at final histopathology were excluded from this analysis.

All the procedures of the study were in accordance with the ethical standards of the Helsinki declaration. Patients’ written informed consent was provided before therapeutic procedures.

Follow-up time was calculated starting from the date of IOERT to the last clinical visit. The outcome variables were acute and late toxicity, cosmetic result, “in-field” local recurrence (in-field LR), “out-field” local recurrence (out-field LR), DFS and OS.

Acute and late toxicity were assessed according to the Common Terminology Criteria for adverse events (CTCAE) (version 4.03) [17] with particular attention to erythema, seroma requiring surgical drainage and infection as acute toxicity, while fibrosis and telangiectasia as late toxicity. The cosmetic results were evaluated by physician (objective) and patient (subjective) according to the Harvard scale [18]. The evaluation was obtained according to the timing of the oncological follow-up.

Clinical examination was performed in all the patients before the start of EBRT, at the end of EBRT, at 3, 6 and 12 months thereafter, and once a year for the next 5 years.

## 3. Statistical Analysis

A statistical analysis of the population was carried out for the evaluation of the oncological endpoints. The outcome variables were acute and late toxicity, cosmetic result, “in-field” local recurrence (in-field LR), “out-field” local recurrence (out-field LR), DFS and OS. Clinical variables, toxicities and cosmetic results were considered as categorial variable and summarized as proportion. In-field LR, out-field LR, DFS and OS were considered as time to event variables. The in-field LR was calculated from the date of IOERT to the time of recurrence in proximity of tumor bed, or last follow-up date. The out-field LR was calculated from the date of IOERT to the time of recurrence in the same breast off tumor bed, or last follow-up date. DFS was calculated from the date of IOERT to the time of LR or presence of distant metastases, or last follow-up date. The OS was calculated from the date of IOERT to death for any cause, or last follow-up date. The Kaplan-Meier method was used to evaluate time to event variables. Data management and statistical analysis were conducted using the open-source R platform (version 3.5.2, The R Foundation for Statistical Computing, Vienna, Austria).

## 4. Results

From January 2011 to March 2018, 797 patients with a median age of 58 years (range: 21–84 years) underwent anticipated IOERT-boost during breast-conserving surgery followed by WBI. In 7 women, EBRT was not performed for individual reasons: refusal of patients in 4 cases, systemic progression in 2 patients, excessively long interval due to intercurrent trauma in one patient). The final analysis was performed in all the patients, considering “the intention to treat”.

Patient and tumor characteristics are shown in Table 2.

The study population received the IOERT-boost to a prescription dose of 9–12 Gy at the reference isodose, and according to the time schedule established by the oncological program, WBI was administrated to a conventional total dose of 50 Gy in 25 fr or with hypofractionated scheme (40.5 Gy/15 fr or 42.56 Gy/16 fr) with 3D-conformal radiation therapy (3D-CRT) technique and different energy photons (6–10 MV), as reported in Table 3. The median time between IOERT-boost and WBI was 6 weeks (range 4–8 weeks). As far as systemic therapy, at 4–15 weeks after surgery, 185 patients underwent adjuvant chemotherapy and 647 patients underwent hormonal therapy, including 126 patients who received adjuvant chemotherapy plus hormonal therapy. Neoadjuvant systemic treatment was not allowed.

The evaluation of acute toxicity (CTCAE v. 4.03) at 4–8 weeks after IOERT and at the end of WBI are reported in Table 4. Overall, acute toxicity was mild and, in fact, no toxicity data higher than grade 3 were reported in any case. A total of 239 (29.99%) patients had no events and most of the adverse events after surgery were mild according to the CTCAE v. 4.03, such as asymptomatic G1 seroma and hematoma in 378 patients (47.43%).

Acute toxicity ≥ G2 after IOERT occurred in 179 patients (22.46%) who developed seroma requiring drainage (G2 which delayed the time of WBI), and only one patient (0.12%) developed surgical wound infection (G3). No wound dehiscence with the need for surgical intervention was documented. There were no grade 4 complications after surgery.

At the end of WBI, most of the patients (73.9%) showed G1 acute toxicity (mild erythema), no G3 were reported.

Late toxicity at 12 months from the end of radiotherapy was focused on the evaluation of the degree of fibrosis which correlates with the symmetry of the breast profile and the presence of telangiectasias in the IOERT area, as shown in Table 5.

In all cases, data regarding cosmetic outcome were assessed at 1 year from the end of radiotherapy, by both physician and patient, and summarized in Table 6. The cosmetic results reported by physician scored as excellent/good in most of the cases (80.18%). The cosmetic results reported by patient were excellent/good and fair in 30.37% and 68.75% of cases, respectively.

At median follow-up time of 57 months (range: 2–109 months), 785 patients were alive and 12 patients had died (10 of tumor and 2 of other causes).

Overall, 13 patients (1.63%) presented local recurrence (LR): 6 patients (0.75%) showed a “true recurrence” in the IOERT field (in-field LR) and 7 (0.88%) patients showed a recurrence outside the treatment field (out-field LR). Twenty-five patients developed distant metastases (3.14%).

**Figure 1:**
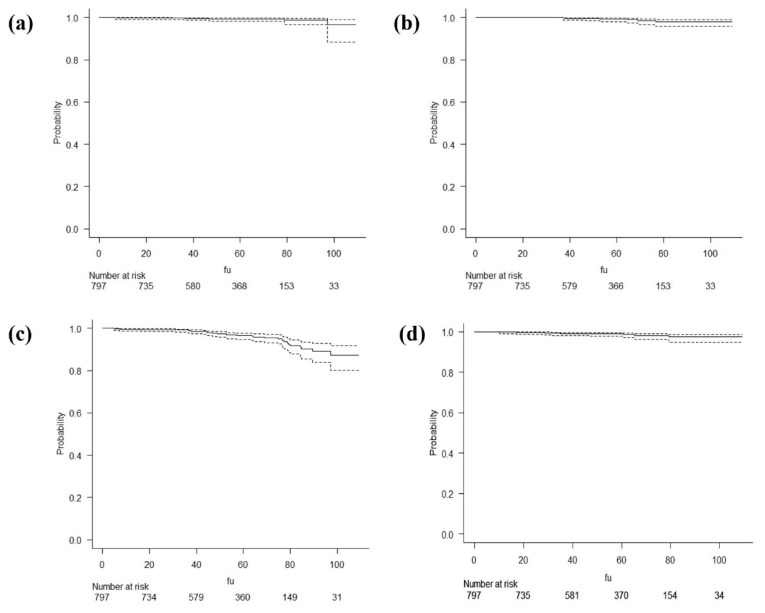
Kaplan-Meier curves for in-field Local Control (**a**), out-field Local Control (**b**), Disease Free Survival (**c**) and Overall Survival (**d**). The dashed lines represent 95% confidence interval in each curve. X axis represents the probability of the event and Y axis the variable time to the event.

At 5 years, in-field and out-field LC were 99.2% (95% confident interval [CI]: 98–99.7) and 98.9% (95% CI: 97.4–99.6), respectively; DFS and OS at 5 years were 96.2% (95% CI: 94.2–97.6) and 98.6% (95% CI: 97.2–99.3), respectively. The Kaplan-Meier curves for the established time to event endpoints are shown in Figure 1.

## 5. Discussion

The loco-regional treatment of breast cancer has radically changed over the past three decades thanks to the evolution of new techniques and technologies aimed at achieving the best results in terms of local control and cosmetic outcome with improved patient survival and quality of life. Since 1990, conservative surgery and adjuvant WBI have been the standard of care for early-stage breast cancer [3]; this approach was shown to achieve good local control with satisfactory cosmetic results while preserving the mammary gland.

Over the years, evidence from randomized studies has led to moderate hypofractionation becoming the new standard of care for whole breast radiotherapy, with data on its efficacy and tolerability comparable to conventionally fractionated radiotherapy, with advantages also in terms of cost and convenience for the patient [19]. Recently, new ultra-hypofractionated WBI schemes have been tested: FAST and FAST-Forward [20,21]. These studies implemented knowledge on the radiation biology of breast cancer and provided clinical results related to late recurrence and side effect events. Both studies confirmed that the α/β value for breast cancer is between 3.5 and 4 Gy, which is comparable to the α/β value for most of the late effects on normal tissues [19].

Usually, WBI is followed by a boost to the tumor bed, which is regarded as the site with the highest probability of recurrence, with either electrons, photons, or brachytherapy [8], and recently, as an integrated simultaneous boost [22,23].

Historically, tumor bed boost has become popular after the publication of the Lyon and Budapest studies [24,25]. In both studies, at the end of the WBI, a dose of 10 and then 16 Gy was able to increase local control especially for women at higher risk of local recurrence. The EORTC 22881-10882 trial is one of the most important studies evaluating the role of the boost; 5569 women were randomized to WBI (50 Gy) or WBI plus a 16 Gy boost to the tumor bed [26]. After a median follow-up of 5.1 years, the results showed a clear benefit of the additional dose in terms of local control, regardless of systemic adjuvant therapy, and the benefit was more evident for patients ≤ 40 years of age. In a more recent update with 20 years of follow-up, the boost was confirmed to improve local control, at the cost of a higher risk of developing moderate fibrosis [6]. In the Young Boost study by Brouwers et al., patients aged >50 years were randomized to receive a boost with a dose of 16 Gy vs. 26 Gy, using photons (73% vs. 74%), electrons (22% vs. 18%) or interstitial brachytherapy (1%) [27]. The evaluation of the cosmetic result was carried out by both medical doctors and patients and turned out to be significantly worse in patients with high-dose boost at 4 years of follow-up. Furthermore, the authors described a significant correlation between the degree of fibrosis and cosmetic outcomes.

In this scenario, the correct identification of the area defined as the “tumor bed” is extremely important to optimize the effectiveness of the treatment, especially in the era of oncoplastic surgery, in which the initial site of the neoplasm may not correspond to that identifiable in the radiotherapy procedures. Various techniques have been suggested for boost planning, but a correct definition of the target remains one of the most important controversies. The identification and treatment of a clinical target volume (CTV) based on the surgical scar can lead to a significant undertreatment of the tumor bed, with implications for local control [28]. Computed tomography images may help in the localization of the area to be treated, with significant variability among observers, especially when a seroma is not clearly visible [29]. In addition, the volume of the excision site tends to change during WBI, thereby adding uncertainty that can only be solved by increasing CTV margins [29,30] and positioning surgical clips in the tumor bed. The use of IOERT-boost can solve the issues related to the correct identification of the target; indeed, the direct visualization of the tumor bed allows a rapid, direct and precise localization of the area to be treated, thereby reducing the possibility of a geographical miss [9,31].

Furthermore, the results of a pilot study conducted on 50 women treated with IOERT-boost (9–20 Gy) followed by WBI (50 Gy, 2 Gy/fr), showed a cosmetic score from good to excellent in all the patients assessed after a median follow-up of 9.1 years [32].

Another important issue is that, assuming an alpha/beta value of 4 for breast cancer, a dose of 10 Gy in one fraction corresponds to approximately 23.3 Gy in an equivalent dose of 2 Gy per fraction, which is more than double compared to the external beam boost. As shown in our results and according to the Brouwers study [27], this dose could be sufficient to ensure control of the disease without compromising the cosmetic result.

Several studies [32,33,34,35,36,37,38,39,40,41,42] have investigated the efficacy of IOERT-boost on local control. Many of them are reported in Table 7.

In the preliminary Salzburg’s experience [43], 190 patients with T1–T2 breast cancer underwent IOERT with a dose of 9 Gy, followed by WBI (51–56 Gy); another group of patients with the same characteristics was treated with a fractionated external boost, with a median follow-up of 25.8 months. There were no cases of LR in the IOERT arm compared to 8 cases in the non-IOERT one (*p* = 0.082), while there was a distant metastases rate of 1.1% in the intra-operative boost group vs. 7.9% in the non-IOERT group (*p* = 0.09). The authors concluded that IOERT boost after breast-conserving surgery appears to be superior compared to the conventional boost in a short-term follow-up. These results were confirmed in a subsequent study on a larger sample size, promoted by the International Society of Intra-operative Radiation Therapy (ISIORT) [38]. The ISIORT analysis included 1109 non-selected patients from 7 different centers using the same doses for IOERT and WBI: 10 Gy at 90% reference isodose for the IOERT boost and 50–54 Gy (1.7–2.0 Gy/fr) for WBI. At a median follow-up of 72.4 months (range 0.8–239 months), 16 breast recurrences occurred and a tumor control rate of 99.2% was achieved.

In addition to these clinical results, another major advantage of IOERT is its radiobiological potential benefit. Indeed, many findings in the field of tumor microenvironment have been acquired. High-dose irradiation not only directly damages the DNA, while it could also promote the immunity of anti-tumor T cells and the expansion of activated T cells [44] as well as target the inflammatory wound response immediately after surgery, thus impacting on the formation of pathways activated by the inflammatory wound response in residual breast cancer cells and the breast microenvironment [45].

In the recently published data from the Salzburg group [41], 10-year results in a non-selected cohort of 770 breast cancer patients were analyzed in terms of LC and survival. Patients were treated with breast-conserving surgery, IOERT (dose: 10 Gy) and WBI (total median dose: 54 Gy). After a median follow-up of 121 months, 21 breast recurrences were observed (2.7%), 107 patients died (14%) and 106 developed DM (14%). Ten-year rates of LC, locoregional control (LRC), metastasis-free survival (MFS), OS and specific breast cancer survival (BCSS) amounted to 97.2%, 96.5%, 86%, 85.7% and 93.2%, respectively. In multivariate analysis, HER-2 positive and triple negative subtypes were significant negative predictive factors for ipsilateral breast cancer relapse (IBR). Surprisingly, and unlike previous analysis of the same cohort, no higher risk of IBR was observed for high grade (G3) tumors and positive lymph node status. After long-term follow-up, IOERT consistently provided high LC rates in patients with stage I–III breast cancer, with a prevalence of LR in the HER-2 positive and triple negative subtypes.

Another phase II study by Ivaldi et al. [37] explored the effectiveness of IOERT as a boost (12 Gy) combined with hypofractionated EBRT (2.85 Gy in 13 daily fractions). The authors evaluated radiation toxicity as a primary endpoint on 204 patients, at a median follow-up of 11 months (range 6–14.6). Acute skin toxicity at the end of treatment was G0–G2 in 96.2% (97.8% in the boost area) and G3 in 3.8% (2.2% in the boost area).

In the long-term follow-up of a randomized study comparing IOERT versus EBRT boost in early-stage breast cancer [42], 245 patients were randomized between IOERT (133) and EBRT (112). The median follow-up was 12 years. The cumulative risk of IBTR at 5–10 years was 0.8% and 4.3% after IOERT boost, compared to 4.2% and 5.3% after EBRT boost (*p* = 0.709). All the IOERT arm recurrences were observed at more than 100 months’ follow-up, whereas the mean time to recurrence in the EBRT group was less (55.2 months) (*p* < 0.05). There was a significantly better cosmetic result for IOERT both in physician and patient evaluations with the greatest difference at the end of EBRT (*p* = 0.006 objective and *p* = 0.0004 subjective).

The first results of the HIOB protocol published recently [46] displayed promising data. After a median follow-up of 45 months (range 0–74), on 583 patients treated with IOERT boost (11.1 Gy) followed by moderate hypofractionated WBI (40.5 Gy/15 fr), acute skin toxicity of grade 0/1 (according to CTCAE-scoring system Version 2) was present in 91% (end of treatment) and 92% (4 weeks later) of cases and late toxicity grade 0/1 (according to LENT-SOMA criteria) in 92.7% (range: 89–97.3%) at 4/5 months and 96.5% (range: 91–100%) at 6 years. Baseline cosmesis after IOERT and before EBRT was excellent/good in 86% of cases (subjective evaluation) and in 74% (objective evaluation).

It should be noted that in our experience, the use of a boost has been extended to patients in which it could currently be omitted. This was mainly due to the historical time of patient accrual, which largely precedes the publication of the long-term results of the EORTC trial defining the benefit of breast boost especially in patients younger than 40 years [6]. In fact, in our analysis, no recurrences have occurred in young patients (<45 years of age) during the long follow-up, confirming that IOERT-boost has an important role in this group of patients [46].

A further consideration is that in G3 tumors, which is one of the main prognostic factors influencing local recurrence [41], local control was even better. This result, although in a limited number of patients, could be relevant in terms of choosing a high single dose on the tumor bed immediately after surgical removal.

The IOERT procedure increased the surgical time by approximately 15–20 min. No major or minor postoperative complications related to IOERT or surgical procedures were observed. Data concerning the early and late side effects are comparable to those found in the literature; no cardiac or pulmonary toxicity were observed. One advantage of IOERT-boost was the reduction of the total treatment time, which had important implications not only for biological but also for economic reasons: in fact, saving at least five fractions per patient led to a significant shortening of the waiting list in the radiotherapy departments. The results of this experience seem to indicate the safety and effectiveness of the treatment with IOERT-boost in terms of local control, overall survival and cosmetic outcome, with a median follow-up of 57 months.

However, this study has some limitations, which should be taken into account. First, it is a retrospective analysis without any patient selection and homogeneity; moreover, it is a large multicenter data collection with predictable inhomogeneity in the techniques and in the standardization of treatment scheduling for both IOERT and WBI. For instance, it should be noted that 9 patients underwent the IOERT treatment as a boost despite the important lymph node commitment (N3), which, unlike the use of exclusive single dose IOERT, does not represent a contraindication to overdose the surgical bed.

Furthermore, due to the characteristics of the collected data, it was impossible to reconstruct exactly the number of patients who performed EBRT concomitantly with chemotherapy, while, as suggested by clinical practice, all patients underwent hormone therapy concurrently with radiotherapy. The third criticality detected in this report concerns the evaluation of the cosmetic result, which, unlike other authoritative works (HIOB trial), showed a subjective evaluation after IOERT-boost that was worse than the objective one. This result can be linked to several factors. First, it is necessary to consider the possible bias of follow-up heterogeneity due to retrospective and multicenter analysis. A further consideration is that, in our work, the cosmetic outcome was assessed at 1 year from the end of radiotherapy and the results were not much different than the those obtained in Ciabattoni et al. [42]; on the contrary, the preliminary results of the HIOB trial showed better baseline cosmesis in the subjective valuation after IOERT and before EBRT [46].

Overall, the main strengths of this analysis are the size of the examined population and the length of the follow-up.

The results are in line with the literature data, thereby confirming the efficacy of the IOERT-boost compared to the EBRT-boost.

## 6. Conclusions

Intra-operative electron boost can be a favorable approach for breast cancer patients requiring adjuvant radiotherapy, as it shortens the total treatment time and post-treatment toxicity. In our experience, it appears to be safe, providing slightly better, although not significant, local control for patients with early-stage tumors. Prospective studies with long follow-up are advisable to confirm these results.

An anticipated boost delivering a high dose in the “right time” and in the “the right site” could reduce relapse events over time.

We believe that the safety and long-term efficacy of IOERT-boost compared to the EBRT-boost should encourage this modality of treatment, with the potential to improve local control of disease and quality of life.

## Figures and Tables

**Table 1:** Radiotherapy centers participating in the study and patients analyzed.

Italian IORT Centers: 8	Patients: 797
San Filippo Neri Hospital, ASL Roma 1, Rome	34 patients
Città di Castello Hospital, Città di Castello-Perugia	411 patients
Salvatore Maugeri Foundation, Pavia	86 patients
Ferrara University Hospital, Arcispedale S. Anna, Ferrara	74 patients
Trieste University Hospital, Trieste	75 patients
Rionero in Vulture Referral Cancer Centre-CROB, Rionero in Vulture–Potenza	59 patients
University Hospital Maggiore della Carità, Novara	14 patients
Bari Cancer Institute, Bari	44 patients

**Table 2:** Patient and tumor characteristics of the study population.

Patient and Tumor Characteristics	
**Total patients**	797
**Median age in years (range)**	58 (21–84)
**Performance status ECOG scale**	**Number (%)**
0	754 (94.61)
1	41(5.14)
2	2 (0.25)
**Laterality**	
Right	389 (48.81)
Left	408 (51.19)
**Histology**	
Invasive ductal carcinoma (IDC)	713 (89.46)
Ductal carcinoma in situ (DCIS)	2 (0.25)
Invasive lobular carcinoma (ILC)	82 (10.29)
**Tumor Stage**	
Tis	2 (0.25)
T1	721 (90.46)
T2	72 (9.04)
T3	2 (0.25)
**Node Stage**	
N0	642 (80.55)
N1	134 (16.81)
N2	12 (1.51)
N3	9 (1.13)
**Grading**	
G1	159 (19.95)
G2	443 (55.58)
G3	195 (24.47)
**Estrogen Receptor**	
Positive	710 (89.08)
Negative	87 (10.92)
**Progesterone Receptor**	
Positive	698 (87.58)
Negative	99 (12.42)
**Human Epidermal Growth Factor Receptor 2 Expression**	
Positive	141 (17.69)
Negative	656 (82.31)
**Ki-67**	
<20%	586 (73.53)
>20%	211(26.47)
**Hormone Therapy**	
Yes	647 (81.18)
No	88 (11.04)
Not specified	62 (7.78)
**Chemotherapy**	
Yes	185 (23.21)
No	548 (68.76)
Not specified	64 (8.03)
**Hormone Therapy Plus Chemotherapy**	126 (15.8)

ECOG–Eastern Cooperative Oncology Group.

**Table 3:** Intra-Operative Electron Radiation Therapy (IOERT) and Whole Breast Irradiation (WBI) doses and patient distribution.

IOERT (Dose)	Patients Number (%)
9 Gy	71 (8.91)
10 Gy	625 (78.42)
11.1 Gy	23 (2.89)
12 Gy	78 (9.78)
**WBI**	**Patients Number (%)**
Conventional External Beam RT	602 (75.5)
Hypofractionated External Beam RT	188 (23.6)
Not performed	7 (0.9)

**Table 4:** Acute toxicity after Intra-Operative Electron Radiation Therapy (IOERT) and after Whole Breast Irradiation (WBI) according to Common Terminology Criteria for Adverse Events (CTCAE) version 4.03.

Acute Toxicity after IOERT	Number (%)
G0	239 (29.99)
G1	378 (47.43)
G2	179 (22.46)
G3	1 (0.12)
**Acute Toxicity after WBI**	**Number (%)**
G0	156 (19.57)
G1	589 (73.9)
G2	52 (6.53)
G3	0

**Table 5:** Late toxicity after Intra-Operative Electron Radiation Therapy and Whole Breast Irradiation (IOERT + WBI) according to Common Terminology Criteria for Adverse Events (CTCAE) version 4.03.

Fibrosis	Number (%)
Yes	336 (42.16)
No	461 (57,84)
**Telangiectasia**	**Number (%)**
Yes	1 (0.13)
No	796 (99.87)

**Table 6:** Objective and subjective cosmetic outcome after Intra-Operative Electron Radiation Therapy and Whole Breast Irradiation (IOERT + WBI) according to the Harvard scale.

Cosmetic Outcome	Objective Number (%)	Subjective Number (%)
Excellent	360 (45.2)	80 (10)
Good	279 (35)	162 (20.3)
Fair	158 (19.8)	553 (69.4)
Poor	0 (0)	2 (0.3)

**Table 7:** Main studies on the treatment with IOERT-boost followed by whole breast radiotherapy.

Author/Year	Follow-Up	*n*	Stage	IOERT (Gy) (Isodose, %)	WBI (Gy)	Local Control (%)	Overall Survival (%)
Merrick et al. 1997 [33]	71 months	21	I–II	10–15 (100)	45–50	Crude 100	Crude 90.5
Dubois et al. 1997 [34]	Min. 24 months	102	I–II	10 (90)	45	Crude 100	-
Lemanski et al. 2006 [32]	109 months	50	I–II	9–20 (90)	50	Crude 96	-
Ciabattoni et al. 2004 [35]	-	234	I–II	10 Gy (100)	50	Crude 100	-
Reitsamer et al. 2006 [36]	51 months (IORT) 81 months (EBRT)	190 (IORT) 118 (EBRT)	I–II	9 (100) (IORT) 12 (EBRT)	51–56	Act. 5 y 100 (IORT) Act. 5 y 95.7 (EBRT)	-
Ivaldi et al. 2008 [37]	8.9 years	204	I–III	13.3 (100)	37.05	Act. 9 m 100%	-
Fastner et al. 2013 [38] (ISIORT)	72.4 months	1109	I–III	6–15 (100)	50–54	Act. 6 y 99.2	Act. 6 y 91.4
Fastner et al. 2015 [39]	59 months (IORT) 67.5 months (EBRT)	83 (IORT) 26 (EBRT)	I–III (preop CT)	9 (100) (IORT) 12 (EBRT)	51–57	Act. 6 y 98.5 (IORT) Act. 6 y 88.1 (EBRT)	Act. 6 y 86.4 (IORT) Act. 6 y 92 (EBRT)
Fastner et al. 2016 [40]	97 months	71	I–II	7–12 (100)	54 med	Act. 8 y 89	Act. 8 y 75
Kaiser et al. 2018 [41]	121 months	770	I–III	5–12 (100)	54 med	Act. 10 y 97.2	Act. 10 y 85.7
Ciabattoni et al. 2021 [42]	12 years	245 (133 for IOERT and 112 for EBRT)	I–III	10 (90)	50	IBTR at 5–10 years was 0.8% and 4.3% after IOERT 4.2% and 5.3% after EBRT boost	OS at 5 and 10 years was 94.5% and 91.6% for IOERT, 99% and 94.3% for EBRT boost

WBI: whole breast irradiation; IOERT and IORT: intra-operative electron radiotherapy and intra-operative radiotherapy; EBRT: external beam radiotherapy; y: years; IBTR: in breast true recurrence: med: median; act: actuarial.

## Data Availability

The datasets used and/or analyzed during the current study are available from the corresponding author on reasonable request.
